# Chloral Hydrate in Obstetrics

**Published:** 1879-03-20

**Authors:** L. B. Bouchelle

**Affiliations:** Georgia


### *CHLORAL HYDRATE IN OBSTETRICS.*

BY L. .B BOUCHELLE, M.D., OF GEORGIA.

There is no article in the materia medica but has its uses and abuses, and this is eminently true of this important drug. The good and great Dr. Dickinson was accustomed to say of opium : "Magnum donum Dei homini"; and as much may truly be said of chloral hydrate. But its special use in obstetrics is all that is proposed to consider in this humble contribution.

No working physician can recall his cases for, say five years, without remembering with mortification, not to say perplexity, how he has been disappointed with ergot in cases of inertia of the womb. But after making due allowance for idiosyncrasis, these will all disappear with the use of chloral. I do not remember to have seen this property of the drug named in any book or journal; and it may be proper to state that it was accidentally noticed in my practice, thus: A case of considerable duration that occurred some years ago, in which I proposed to help the sufferer to a little respite, by giving a moderate dose of the drug; and, instead of accomplishing my purpose, it set the pain to "grinding" with redoubled vigor, and ended in a far more speedy recovery than had been anticipated. After that it was administered again and again to test this property with most gratifying results, since which time ergot has been appealed to but once. And in addition to its superiority in exciting uterine action to ergot, after delivery, it soothes the sufferer into a refreshing "nap," from which she awakes feeling fresh and bright. Is the rationale asked for by the positivist? Let him tell us why some drugs excite one organ to action and not another, and it will be time to explain this.

Some years ago the writer had occasion to write for this journal some account of treating puerperal convulsions, without blood letting, substituting brisk catharsis; now by the use of chloral, I have no trouble in controlling this fearful disease, either *before* or *after* delivery. Under the old regime, in case the convulsions came before delivery, this was the first thing thought of; but, as seems more natural and most demanded, by the exigences of the case, with chloral the convulsions are stopped at once, and an easy and natural delivery is facilitated.

In such cases it acts equally as well or better administered per anum, or per orem, without much increase of dose. Only be careful to use enough of fluid to keep from irritating the bowel too much, but not enough to so distend it as to provoke peristaltic action. It is better to use sugar, glycerine or albumen with the water. The writer has never had to give more than the second dose without completely subduing the convulsions. In only one case has he found it necessary to resort to any other remedy whatever in a number of cases.

This remedy has several distinctive features that commend it to the profession. First, its simplicity; second, its certainty; third, its innocency with its promptness, and last but not least, its dual property: it subdues the convulsions and facilitates delivery, when they occur *ante partum*.

From my book cases might be given seriatim, but to state well-established facts, in as small compass as possible, is my object in this communication, and to give my peers the benefit of my experience in a disease that may well strike us with terror, for it is a terrible malady. I give, to arouse to energetic action, the uterus that is inert, 20 to 30 grains, in, say one gill of water made pretty sweet, and have never had to repeat. Who can say as much for ergot? In convulsions the same dose, per anum, in about the same quantity of water, sweetened, or with glycerine, or the white of an egg well mixed with the water. In such cases, the second dose is rarely needed.

A convulsion in a family is like a shell in a camp, it puts dismay on every countenance; but with this potent remedy the doctor can go to the rescue with *almost unlimited confidence*, if these miserable torturers have not had hold of the patient long enough to shake the vitality out of her, or so near out, that the tide ebbs on after the cessation of the convulsions. Try it.

